# Inhibiting Glutathione Metabolism in Lung Lining Fluid as a Strategy to Augment Antioxidant Defense

**DOI:** 10.2174/157340811796575308

**Published:** 2011-07

**Authors:** Martin Joyce-Brady, Jun Hiratake

**Affiliations:** 1 The Pulmonary Center, Boston University School of Medicine, Boston, MA 02118, USA; 2 Institute for Chemical Research, Kyoto University, Gokasho, Uji, Kyoto 611-0011, Japan

**Keywords:** Lung lining fluid, glutathione, metabolism, antioxidant, γ-glutamyl transferase.

## Abstract

Glutathione is abundant in the lining fluid that bathes the gas exchange surface of the lung. On the one hand glutathione in this extracellular pool functions in antioxidant defense to protect cells and proteins in the alveolar space from oxidant injury; on the other hand, it functions as a source of cysteine to maintain cellular glutathione and protein synthesis. These seemingly opposing functions are regulated through metabolism by gamma-glutamyl transferase (GGT, EC 2.3.2.2). Even under normal physiologic conditions, lung lining fluid (LLF) contains a concentrated pool of GGT activity exceeding that of whole lung by about 7-fold and indicating increased turnover of glutathione at the epithelial surface of the lung. With oxidant stress LLF GGT activity is amplified even further as glutathione turnover is accelerated to meet the increased demands of cells for cysteine. Mouse models of GGT deficiency confirmed this biological role of LLF GGT activity and revealed the robust expansiveness and antioxidant capacity of the LLF glutathione pool in the absence of metabolism. Acivicin, an irreversible inhibitor of GGT, can be utilized to augment LLF fluid glutathione content in normal mice and novel GGT inhibitors have now been defined that provide advantages over acivicin. Inhibiting LLF GGT activity is a novel strategy to selectively augment the extracellular LLF glutathione pool. The enhanced antioxidant capacity can maintain lung epithelial cell integrity and barrier function under oxidant stress.

## LUNG LINING FLUID (LLF)

Recent research using mouse models susceptible to oxidant stress supports a cause and effect relationship between antioxidant defense and susceptibility to lung injury [1]. These studies, together with others on acute inflammatory lung disease [2, 3] have renewed interest in glutathione homeostasis as an antioxidant defense mechanism within the lung. The lung is lined by a continuous, thin layer of fluid (lung lining fluid, LLF) that bathes the entire epithelial surface. LLF functions at the air-liquid interface as an aqueous medium for exchange of molecules within the surfactant system, a supportive medium for the alveolar macrophage, a protective surface for the thin alveolar septum, and a component of the air-blood diffusion distance [4]. In its protective function LLF shields cells against oxidants, which may be inhaled from the environment or generated endogenously by inflammatory cells. Glutathione is one of the small antioxidant molecules within LLF. Although some argue that it is not the most abundant of these antioxidants, LLF glutathione has been the focus of much attention for at least two reasons: its concentration exceeds that of blood by over 100-fold, and its major form is the antioxidant glutathione (GSH), as opposed to glutathione disulfide (GSSG) [5]. Glutathione is a versatile antioxidant within the LLF where it also coats the entire lung surface [6]. It directly buffers the hypohalous acids, very potent and potentially injurious oxidants produced by inflammatory cells [7] and with inhalation of chlorine gas [8]. It limits the accumulation of hydrogen peroxides and lipid peroxides indirectly by functioning as a cofactor for extracellular glutathione peroxidase [9]. Glutathione also maintains the bioavailability of small antioxidant molecules, such as nitric oxide [10], ascorbic acid [11], and alpha-tocopherol [12]. More recently, glutathione and glutathione disulfide have been shown to exhibit an added antioxidant activity by their ability to complex with metals, such as Fe^II^ and Cu^I ^[13]. Recent reviews have detailed the role of lung glutathione production in inflammation and lung disease [14, 15]. Our review will focus on the role of the LLF glutathione pool in antioxidant defense at the lung epithelial surface. Herein we will discuss a strategy to augment LLF glutathione content by inhibiting LLF glutathione metabolism. 

## AUGMENTING LLF GLUTATHIONE

While determinants of the exact size of the LLF glutathione pool are not fully understood, augmentation of LLF glutathione has been shown to protect against oxidant-mediated injury [15]. LLF glutathione content has been correlated with susceptibility to and severity of several lung diseases. In asthmatics, where increased oxidant burden is present in the airways due to inflammation, higher levels of LLF glutathione correlate with lower levels of airway hyperresponsiveness in humans [16] and in a mouse model of cytokine-driven allergic airway inflammation [3]. LLF glutathione is increased in smokers with COPD compared to non-smokers [5], but this rise is still less than that of smokers without COPD, suggesting that sufficient augmentation of this pool protects against COPD [17]. In the acute respiratory distress syndrome (ARDS), LLF glutathione deficiency in hospitalized alcoholics has been directly linked with increased incidence and severity of lung injury [18]. Replacement of this deficit can alleviate this risk [19, 20], a biologically relevant correlation to the recent finding that oxidizing events in LLF directly link with acute lung injury pathogenesis [2]. LLF glutathione deficits are also present in idiopathic pulmonary fibrosis (IPF) [21], HIV disease [22] and cystic fibrosis [23]. In fact, increases in the LLF glutathione pool may account for some of the protective effects of hypertonic saline in cystic fibrosis [24]. All together, these observations support a role for LLF glutathione in protection against cell injury and lung disease. Hence continued assessment of LLF glutathione content and development of new strategies to augment this extracellular glutathione pool are warranted. 

Several mechanisms have been investigated to manipulate LLF glutathione content. Others are likely to follow as we learn to manipulate glutathione export and perhaps glutathionylation of LLF proteins [25]. One general strategy involves direct instillation of glutathione through the airway. Prousky has reviewed human trials utilizing this approach [26]. By and large this strategy produces modest increases in glutathione content. Side effects include increased GSSG content over GSH content and induction of bronchospasm [27, 28]. Bronchospasm may have resulted from sulfite sensitivity [26], or failure to neutralize the glutathione solution pH prior to instillation [29].

A second strategy involves modulating glutathione content with supplements of glutathione precursors, such as N-acetylcysteine, Procysteine or S-adenosylmethionine. These can be delivered directly through the airway or systemically. Oral delivery of these agents may have little direct impact on LLF glutathione [30, 31] but there value may still lie in the augmentation of cellular glutathione pools and increasing glutathione availability for export into LLF. Their effectiveness may only be evident when these glutathione pools are actually deficient [19, 20]. The membrane permeable precursor γ-glutamylcysteinylethyl ester has also been successfully used to supplement cellular glutathione content [32] and additional esterified precursors and glutathione itself are described in the literature [33].

A third strategy involves modulating glutathione metabolism by inhibiting GGT activity. This approach was based on our thorough characterization of GGT expression in normal lung, together with an animal model of GGT deficiency, the GGT^enu1^ mouse [34-38]. We found that LLF GGT activity is: 1) associated with surfactant phospholipid, 2) dynamic in nature, 3) directly accessible for pharmacologic manipulation and 4) a potent target for augmenting LLF glutathione content [3]. Limitation in cysteine supply induced by eliminating glutathione metabolism can be readily reversed with an external cysteine supplement. 

A fourth strategy involves delivery of aerosolized secretory leukoprotease inhibitor (SLPI). This protease inhibitor can increase LLF glutathione up to 5-fold and the effect lasts even at 24 hours after application [39]. This finding was totally unexpected and the mechanism of action remains unclear. However, SLPI does not inhibit GGT enzyme activity (R.P. Hughey and M. Joyce-Brady, unpublished observation) and this protease inhibitor could be explored for an additive effect on LLF glutathione augmentation when combined with inhibition of glutathione metabolism. 

## GLUTATHIONE METABOLISM IN LLF 

Glutathione metabolism is regulated by GGT. The protein is synthesized as a monomer but the active enzyme is a heterodimer that is anchored to the external surface of the plasma membrane by its signal sequence [40]. It plays an essential role in the metabolism of extracellular glutathione and its S-conjugates by cleaving the γ-glutamyl amide bond. While the full physiological function of GGT is yet to be completely defined [41-43], as an ectoenzyme, it is believed to at least initiate the hydrolysis of extracellular glutathione to provide cells with secondary source of Cys, which is the rate-limiting substrate for *de novo* synthesis of intracellular glutathione [44, 45]. The enzyme is also present as a soluble form in extracellular biological fluids where it can function to distribute glutathione between cells and tissues [46]. 

The GGT activity found in normal LLF is present in association with lung surfactant phospholipid. This soluble activity is derived, in part, as a secretory product of the alveolar type 2 (AT2) cell, and the amphipathic nature of GGT allows its redistribution throughout the entire surface of the lung along with surfactant [36]. The ontogeny of GGT in the AT2 cell during late fetal lung development parallels that of surfactant phospholipid so that LLF glutathione metabolism is active from the time of birth [37].

The GGT^enu1^ mouse model of genetic GGT deficiency [34, 35] provided support for this biological role of glutathione metabolism in the lung. With limited cysteine availability, lung cells exhibited impaired glutathione synthesis, cellular glutathione deficiency, and oxidant stress in normoxia [47]. This was most evident in bronchiolar Clara cells, alveolar macrophages and vascular endothelial cells. In hyperoxia, cellular glutathione deficiency in the presence of this intracellular oxidant stressor, prediposed to excessive lung injury and accelerated mortality in GGT^enu1^ mice [47, 48]. Dietary supplements with the cysteine precursor N-acetyl cysteine [48, 49] or L-2-oxothiazolidine-4-carboxylate [50] attenuated the cellular glutathione deficiency and lung sensitivity to hyperoxia [48].

**Fig. (1):**
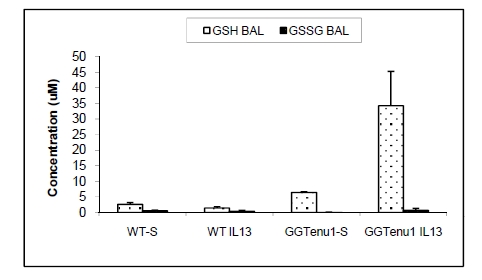
**LLF glutathione (GSH) and glutathione disulfide (GSSG) in normal (wild type, WT) and GGT^enu1^ mice after saline (S) or IL13 treatment.** LLF glutathione assessed as bronchoalveolar lavage fluid (BAL).

However, glutathione content in the extracellular LLF pool of GGT^enu1^ mice with genetic GGT deficiency was actually augmented in a fashion similar to that described in plasma [34, 49]. The increase in this glutathione pool strongly supported the concept that LLF glutathione undergoes turnover in the normal lung. The biological role of this LLF glutathione enhancement became evident when GGT^enu1^ mice were exposed to an IL13-driven model of inflammatory airway disease [3]. Pro-inflammatory IL13 treatment activated an extracellular burden of oxidant stress from the acute inflammatory response. In normal mice, there was little change in LLF fluid glutathione, GSH (Fig. **1**). BAL LLF glutathione in GGT^enu1^ mice started a 2-fold over normal baseline and increased 5-fold more after IL13, a level that was about 10-fold above the baseline level in normal mice. 

**Fig. (2):**
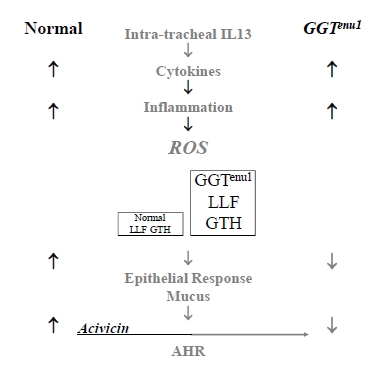
**Loss of GGT activity augments LLF glutathione in presence of IL13.** IL13, a pro-inflammatory cytokine, induces inflammation and an extracellular load of reactive oxygen species (ROS). These are buffered by the surplus of LLF glutathione in GGT deficient GGT^enu1^ mice and injury is prevented. Normal mice are susceptible to injury and can be protected by inhibiting their LLF GGT with acivicin.

This surplus of LLF glutathione buffered extracellular reactive oxygen species derived from inflammatory cells and protected proteins in the LLF and the lung epithelial surface against oxidant stress, epithelial cells from mucin gene induction and airways against hyperreactivity. These were all induced in normal mice treated with IL13 but they could be partially attenuated by inhibiting their LLF GGT activity with the irreversible GGT inhibitor acivicin (Fig. **2**). Interestingly, we found, as had others, that delivery of acivicin systemically had no effect on LLF GGT activity. To effectively inhibit this extracellular pool of enzyme activity and modulate LLF glutathione, acivicin had to be delivered through the airway [3]. 

## INHIBITION OF GGT ENZYME ACTIVITY

**Fig. (3):**
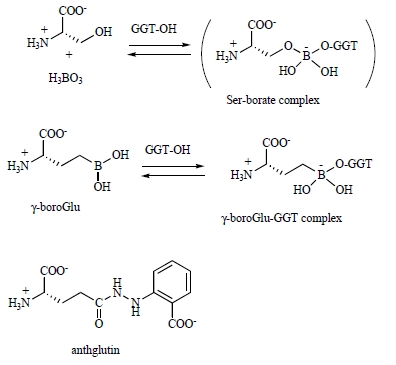
**Classical inhibitors of GGT.** These include: serine-borate complex, γ-boronate analog of glutamate and anthglutin.

**Fig. (4):**
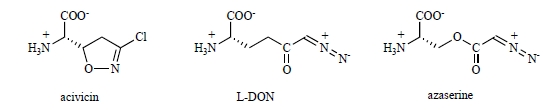
**Naturally occurring glutamine antagonists that inhibit GGT activity.** L-DON: 6-diazo-5-oxo-L-nor-leucine.

While several compounds are known to inhibit GGT enzyme activity, designing novel, potent and more selective inhibitors required a rational and mechanistic understanding of enzyme function. GGT-mediated glutathione hydrolysis occurs by a ping-pong mechanism [51-54] and utilizes a γ-glutamyl ester intermediate (an acylenzyme) with an *N*-terminal Thr residue in the small subunit (Thr391 [55] and Thr381 [56] of *E. coli* and human GGTs, respectively) as the catalytic nucleophile. The γ-glutamyl group is then transferred to water (hydrolysis) or to various amino acids and peptides (transpeptidation) if these acceptor molecules are present in high concentrations. Under physiological conditions, however, GGT is mainly regarded as the hydrolytic enzyme that initiates the release of Cys and other constituent amino acids from extracellular glutathione [44, 45]. For *in vitro* and *in vivo* studies to probe the mechanisms and physiological functions of GGT, a number of inhibitors have been reported to date; the classical inhibitors of GGT include a serine-borate complex [57], a γ-boronate analog of glutamate (γ-boroGlu) [58, 59], anthglutin [60, 61] (Fig. **3**) and several naturally occurring glutamine antagonists (Fig. **4**) [62-67]. 

A serine-borate complex is a transition-state like adduct formed tentatively in the enzyme active site (*K*_i_ = 0.02 mM) and is readily dissociated when the enzyme is dialyzed [57]. Its boronate analog (γ-boroGlu) serves as a slow- and tight-binding inhibitor with a substantial potency (*K*_i_ = 35 nM), but the inhibition is still reversible, and the inactivated enzyme regains activity rapidly [59]. Its development seems to have been terminated, and no further information is available regarding the properties of this compound such as the specificity and toxicity. Anthglutin was screened for GGT inhibitory activity from the culture medium of *Penicillium oxalicum*. Anthglutin is a naturally occurring glutathione analog and serves as a competitive inhibitor for various GGTs with *K*_i_ values of 5-27 µM [60]. No acute toxicity was reported for mice (100 mg/kg of body weight). These classical inhibitors, however, are of limited use for *in vivo* inhibition of GGT in lung lining fluid, because they are rather weak or reversible so they do not suppress GGT activity for an appropriate period of time to exert therapeutic effects.

In contrast, the naturally occurring glutamine antagonists such as acivicin [L-(α*S*,5*S*)-α-amino-3-chloro-4,5-dihydro-5-isoxazoleacetic acid, AT-125], L-DON (6-diazo-5-oxo-L-norleucine) and azaserine (*O*-diazoacetyl-L-serine) are all chemically reactive and inhibit GGT irreversibly [62-67]. Among these glutamine antagonists, acivicin is by far the most popular inhibitor of GGT and has been used extensively not only for *in vitro* experiments [62-64], but also for *in vivo* studies to see the effect of chemical knockdown of GGT on tumor cells [68-71], bacterial pathogenicity [72], signal transduction in myocardial infarction [73], oxidative stress in pulmonary vascular endothelial cells [74] and xenobiotic metabolism in plants [75]. The main reason for the frequent use of acivicin is that this compound is commercially available (ex. Santa Cruz Biotechnology, Inc., USA; Haihang Industry Co., Ltd., China) and reacts readily with the catalytic Thr residue of GGT to form a covalent bond [76, 77]. No regain of enzyme activity was reported. Similarly, other glutamine antagonists such as L-DON and azaserine inhibit GGT in an irreversible manner, although the inactivation potency varies depending on the compound. From a pharmaceutical point of view, however, there is a critical problem associated with toxicity in using these compounds for the inactivation of GGT* in vivo*. Acivicin and the related glutamine antagonists are highly cytotoxic and inhibit a number of glutamine-dependent biosynthetic enzymes [78, 79] such as glutamine PRPP amidotransferase, FGAR amidotransferase, IGP synthase, GMP synthetase and carbomoylphosphate synthetase involved in *de novo* purine and pyrimidine biosynthesis. Glucosamine 6-phosphate synthase, asparagines synthetase, NAD synthetase and anthranilate synthase are also inactivated by these compounds [80]. Acivicin is reported to have central nervous system (CNS) toxicity [81]. The toxic nature of acivicin and the related glutamine antagonists is based on a common mechanism: alkylation of the conserved and catalytically essential Cys residue of the glutaminase domain of the amidotransferases by the chemically reactive imino chloride (acivicin) [82-84] and diazoacyl groups (L-DON and azaserine) [79]. In this sense, these glutamine antagonists can be regarded as naturally occurring inhibitors of glutamine amidotransferases that liberate ammonia from glutamine for use as a nitrogen source of nucleotides, amino acids and amino sugars, but not *per se* as inhibitors of GGT. Therefore, the inactivation of GGT by acivicin, L-DON and azaserine is a fortuitous event that derives from the fact that GGT has a nucleophilic and catalytically essential Thr residue at the binding site near the γ-carboxy group of glutamine derivatives such as glutathione. 

**Fig. (5):**
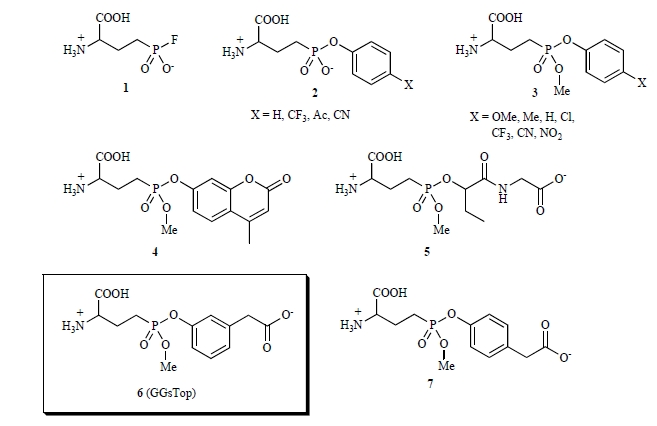
**Novel, potent γ-phosphono diseter analogs of glutamate.** Each compound is referred to by number in the text.

In our effort to identify the catalytic residue of GGT, a γ-monofluorophosphonate derivative of glutamate (Fig. **5**:****compound** 1**) was synthesized. This compound served as a potent inactivator of *E. coli* GGT as a transition-state analog for successful affinity labeling the *N*-terminal Thr-391 in the small subunit as the catalytic nucleophile [55]. The fluorophosphonate (**1**), however, is chemically too reactive to be used as a general inhibitor of GGT. To attenuate the reactivity, a series of γ-(monophenyl)phosphono glutamate analogs were synthesized [85]. These compounds irreversibly inhibited *E. coli* and human GGTs with a reasonable rate and the inactivation rates toward the human enzyme surpassed that of acivicin when an electron-withdrawing group was introduced (X = Ac, CN). In fact, compound **2** (X = CN in Fig. **5**) was used successfully to identify the catalytic nucleophile of human GGT (the N-terminal Thr-381 in the small subunit) [56]. In line with this study targeting the GGT catalytic nucleophile and the glutamate binding site, a series of γ-phosphono diester analogs of glutamate (**3**) were synthesized as second-generation inhibitors [86]. Due to the increased electrophilicity of the phosphorus, these phosphonate diesters are 20 to 40-fold more active than the corresponding monoesters (**2**). To our surprise, the umbelliferone derivative (**4**) exhibited an extraordinarily high activity toward the human enzyme with the inactivation rate reaching 6000 times that of acivicin. This finding has led to design the third-generation inhibitors (**5** and **6**) which mimic the structure of glutathione and its C-terminal carboxy group to interact with the active site of the human enzyme. In particular, compound **6** with a simplified Cys-Gly moiety (a phenyl ring) and a carboxymethyl group at the *meta* position is highly promising in that it compromises chemical stability and high activity toward the human enzyme. In fact, compound **6** is reasonably stable (3% hydrolysis in neutral water for 1 month at 25°C) and inhibited human GGT with an inactivation rate more than 120 times higher than that of acivicin. No regain of enzyme activity was observed. Interestingly, the *para*-substituted analog (**7**) was 155-times less active than compound **6**, indicating that the human enzyme strictly recognizes the *meta*-carboxy group that is equivalent to the C-terminal carboxy group of glutathione[86]. Furthermore compound **6** does not inhibit glutamine-dependent asparagines synthetase, has no toxicity toward human fibroblasts up to 10 mM and has passed the GLP safety guidelines (unpublished results). Therefore, compound **6** is so far the most promising candidate for pharmaceuticals to chemically inactivate GGT activity *in vivo*. This compound does inhibit lung GGT activity, but like acivicin, our preliminary data shows that it must be delivered through the airway (Joyce-Brady and Hiratake, unpublished observation). Hence the advantages of compound **6** include: specificity, potency, and lack of toxicity. Compound **6** is now commercially available under the name of GGsTop from Wako Pure Chemical Industries, Ltd., Japan.

Other synthetic GGT inhibitors reported to date include a series of L-homocysteine analogs (sulfides, sulfoxides, sulfones and sulfoximines) [87, 88]. A sulfoxide analog with a Cys-Gly moiety exhibited the highest activity (*K*_i_ = 53 µM, competitive with respect to γ-glutamyl 4-nitroanilide) toward rat kidney GGT, highlighting the importance of the binding of the Cys-Gly moiety of glutathione with mammalian GGT.

Recently, Hanigan *et al.* reported a high-throughput screening approach to find a drug-like, non-glutamate analog **OU749** and its derivatives as inhibitors of human GGT [89]. From kinetic studies, this compound was found to occupy the acceptor site of the γ-glutamyl substrate complex with a *K*_i_ of 17.6 µM. The inhibition is species-specific and is reported to inhibit human kidney GGT with 7- to 10-times potency than those from rat or mouse kidney. Despite the fact that these compounds target the acceptor-site of GGT, toxicity is still reported. Optimal activity also requires a high level of GGT enzyme activity which is not the case in the lung [36].

## CONCLUSIONS

LLF is composed of a highly concentrated pool of glutathione that serves a biological role in antioxidant defense and cysteine supply over the entire lung surface. This extracellular glutathione pool is dynamic and reduced glutathione predominates. That LLF glutathione content is related to the level of oxidant burden at the lung epithelial surface has been recapitulated in studies over time. Ongoing efforts to assess and manipulate this pool to protect against extracellular oxidant stress are warranted and novel mechanisms to accomplish this goal are available. Studies suggest that early identification and correction of LLF glutathione deficiency can prevent lung injury and disease. Glutathione metabolism plays a role in determining the size of the LLF glutathione pool. Induction of GGT activity with the onset of acute lung injury and inflammation contributes to a relative deficiency of LLF glutathione, even in children with cystic fibrosis [90]. Inhibiting this metabolism by targeting active GGT enzyme can bolster LLF glutathione content and augment antioxidant defense at the lung surface. It is remarkable that targeting of a single antioxidant enzyme in LLF can be protective even in the presence of a high inflammatory load [3]. Nonetheless, oxidizing events originating within the LLF have recently been proposed as the unifying mechanism that initiates syndromes of acute lung injury [2] and cytokine-driven asthma [3]. Since LLF proteins are directly accessible to pharmacologic interventions, focused assessment and specific manipulation of LLF glutathione to enhance extracellular antioxidant defense may yet prove to be a viable strategy to prevent and alleviate oxidant-mediated lung injury. 

## Figures and Tables

**Fig. (6):**
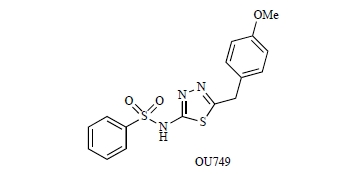
Chemical structure OU749.

## ABBREVIATIONS

GGT: = γ-glutamyl transferase
GSH: = Glutathione
GSSG: = Glutathione disulfide
SLPI: = Secretory leukoprotease inhibitor
GGT^enu1^: = γ-glutamyl transferase deficient mouse mutated with ethylnitrosourea
BAL: = Bronchoalveolar lavage
Thr: = Threonine
DON: = 6-diazo-5-oxo-L-norleucine
PRPP: = Phosphoribosylpyrophosphate
FGAR: = 5-phosphoribosyl-N-formylglycinamide
IGP: = Imidazole glycerol phosphate
GMP: = Guanine monophosphate
NAD: = Nicotinamide adenine dinucleotide
